# A high Diabetes Risk Reduction Score (DRRS) is associated with a better cardio-metabolic profile among obese individuals

**DOI:** 10.1186/s12902-023-01279-5

**Published:** 2023-02-03

**Authors:** Goli Siri, Negin Nikrad, Sheida Keshavari, Saideh Jamshidi, Ehsan Fayyazishishavan, Abnoos Mokhtari Ardekani, Mahdieh Abbasalizad Farhangi, Faria Jafarzadeh

**Affiliations:** 1 Department of Internal Medicine, Amir Alam Hospital, Tehran University of Medical Sciences, Tehran, Iran; 2 Department of Community Nutrition, Faculty of Nutrition, Tabriz University of Medical Sciences, Tabriz, Iran; 3 Echocardiography Research Center, Rajaie Cardiovascular Medical and Research Center, Iran University of Medical Sciences, Tehran, Iran; 4 Department of Biostatistics and Data Science, School of Public Health, The University of Texas Health Science Center at Houston (UTHealth), Houston, TX77030 USA; 5 Endocrinology and Metabolism Research Center, Institute of Basic and Clinical Physiology Science & Physiology Research Center, Kerman University of Medical Sciences, Kerman, Iran; 6 Drug Applied Research Center, Tabriz University of Medical Sciences, Attar Neyshabouri, Daneshgah Blv, Tabriz, Iran; 7 Department of Internal Medicine, School of Medicine, North Khorasan University of Medical Sciences, Bojnourd, Iran

**Keywords:** Diabetes risk reduction, Metabolic syndrome, Obesity, Metabolic parameters, Diabetes

## Abstract

**Background:**

Dietary indices and scores are valuable predictive markers against chronic diseases. Several previous studies have revealed the beneficial effects of diabetes risk reduction score (DRRS) against diabetes and cancer incidence. However, its association with metabolic abnormalities among obese individuals have not been revealed before. In the current study, we aimed to investigate the association between DRRS and metabolic risk factors among obese individuals.

**Methods:**

In the current cross-sectional study, 342 obese individuals [Body mass index (BMI) ≥ 30 kg/m^2^] aged 20–50 years were included. Dietary intake was assessed by a validated semi-quantitative food frequency questionnaire (FFQ) of 168 food items and DRRS was calculated. Metabolic syndrome (MetS) was defined based on the guidelines of the National Cholesterol Education Program Adult Treatment Panel III (NCEP-ATP III). Enzymatic methods were used to assess serum lipids, glucose, and insulin concentrations. Blood pressure was measured by a sphygmomanometer and body composition with bioelectrical impedance analysis (BIA).

**Results:**

Those with a higher adherence to DRRS had a significantly higher intake of energy, fiber, and lower protein compared with those in the lower quartiles. Moreover, lower intakes of trans fats, meat, sugar sweetened beverages (SSB), and glycemic index (GI) with higher intakes of fruits, cereal fiber, polyunsaturated fatty acids/ saturated fatty acids (PUFA/ SFA) ratio, coffee, and nuts were observed in the highest versus lowest DRRS categories. Lower systolic blood pressure, diastolic blood pressure, triglyceride and, higher high-density lipoprotein values were observed in higher DRRS categories. Logistic regression analysis showed that hypertension was significantly associated with adherence to DRRS among obese individuals, the odds ratio (OR) was 0.686 (95% confidence interval [CI], 0.26–0.84) after adjustment for potential confounders. But the risk of other components of MetS was not significantly associated with higher quartiles of adherence to DRRS. Also, a non-significantly lower prevalence of MetS was observed in the higher quartile of DRRS.

**Conclusions:**

According to the results of the current study, higher DRRS was associated with lower blood pressure, modified serum lipids, and lower Mets prevalence. Further studies in different populations are warranted for better generalization of the obtained findings.

## Introduction

Obesity is one of the most important health concerns in the world and is associated with numerous co-morbidities; obesity is associated with reduced quality of life [1–3], increased risk of mental illness and psychological problems [4–7], alongside with increased weight self-stigma and body image dissatisfaction and distortion [8–10]. Also, obese individuals are at greater risk of non-communicable metabolic diseases like type 2 diabetes [11, 12], metabolic syndrome (MetS) [13, 14], cardiovascular disorders [15–17], kidney problems [18, 19], and most types of cancers [20–22]. Lifestyle modification including changes in dietary behaviors and physical activity schedule is one of the most important preventive and therapeutic approaches against obesity [23–25]; in recent years numerous dietary interventions for obesity treatment have been developed and multiple dietary indices for obesity prevention and prediction of obesity-related disorders have been proposed including dietary quality indices [26, 27], dietary inflammatory index [28], dietary diversity score [29, 30], and dietary antioxidant capacity [31], or special dietary regimens (e.g. Mediterranean dietary pattern [32], or MIND diet [33], etc.

These dietary indices are focusing on a particular aspect of diet; for example, dietary antioxidant capacity focuses on the antioxidant potential of diet or dietary diversity score, mostly considers the diversity of food items that one consumes. However, dietary scores that are focusing on the disease-diet associations are most important and useful, because they are taking a direct potential of one’s diet in increasing the risk of a special disease into account; the diabetes risk reduction score (DRRS) is a newly developed index, first introduced by Rhee JJ et al. [34], as a combined score of several dietary components that are recently been found to be associated with risk of type 2 diabetes, these food components include red and processed meats, nuts, coffee and sugar-sweetened beverages (SSBs), glycemic index (GI), cereal fiber, polyunsaturated fatty acid to the saturated fatty acid ratio (P:S), and trans fats. Based on the DRRS components, this diet is generally high in phenolic compounds, antioxidant vitamins and minerals, and antioxidant nutrients, also DRRS contains a lot of unsaturated fatty acids, which may have anti-inflammatory, antioxidant, and anti-atherogenic properties, So adopting a diet with diabetes risk reduction features may lower the risk of developing cardio-metabolic risk factors including insulin resistance [35]. Regarding the DRRS's anti-inflammatory components, the improvement in insulin and leptin sensitivity and decreased inflammation state favor the effects of alpha-melanocyte stimulating hormone (α-MSH) on controlling appetite, increasing satiety, and increasing energy expenditure [36]. Additionally, agouti-related peptide (AgRP) is an endogenous antagonist of α-MSH, and the secretory activity of AgRP neurons is controlled by inflammatory signals [37–39]. A limited number of studies have been performed regarding the DRRS and disease association; such as risk of type 2 diabetes [34], breast cancer, pancreatic cancer, and hepatocellular carcinoma [40–43]. In addition, in a study of Iran, a negative association between higher adherence to DRRS and components of metabolic syndrome has been revealed [35]; again, the same authors identified same results in patients with chronic kidney diseases (CKD) [44]. As mentioned above, there are limited number of studies showing the beneficial effects of higher adherence to DRRS and incident metabolic disorders; and further studies should be performed to identify its possible beneficial effects in different disease statuses. Moreover, obesity is a chronic situation that leads to numerous co-morbidities and it is essential to study the preventive role of diet. By evaluation of the relationship between DRRS and cardio-metabolic risk factors, it can be stated that whether adherence to DRRS with sufficient intake of healthy dietary factors in reduced intake of diabetes-triggering food items can be associated with reduced occurrence of cardiovascular disease risk factors and the consequent prevention of chronic diseases? In the current cross-sectional study, we evaluated the associations between higher adherence to DRRS and cardiovascular risk factors including serum lipids, glycemic markers, and indicators of insulin resistance and inflammatory response among obese individuals.

## Methods and materials

### Participants

The participants of the current cross-sectional study were those who participated in two previous projects, including 342 obese individuals (57.9% males and 41.5% females) [45–47]. Study subjects were invited by public announcements from both Tabriz and Tehran cities and were aged between 20 to 50 years old with a body mass index (BMI) of more than 30 kg/m^2^. The exclusion criteria included: being pregnant, lactating, menopause, having recent bariatric surgery, or any cardiovascular disorders, cancers, hepatic and renal diseases, diabetes mellitus, and taking any weight-affecting medications. Full-informed approved written consent was taken from all of the participants and the study proposal was approved by the Ethics Committee of Tabriz University of Medical Sciences, Tabriz, Iran (registration code: IR.TBZMED.REC.1398.460 and IR. TBZMED.REC.1396.768).

### General characteristics and anthropometric assessments

Socio-demographic information including sex, age, smoking status, education attainment, marital status, occupation, medical histories, and family size were obtained via a questionnaire; then, socioeconomic status (SES) score was calculated [47]. SES was determined using the information about educational status, occupational position, home ownership, and family size. Education was considered as a categorical variable in the current study, while individuals were asked to mention their highest degree of educational attainment. This variable was graded on a 5-point scale ranging from 0 to 5. (Illiterate: 0, less than diploma: 1, diploma and associate degree: 2, bachelors: 3, masters: 4 and higher: 5). The occupational class of female subjects were divided into five categories (housewife, employee, student, self-employed and others). Male individuals' occupational status was classified as follows: 1 unemployed, 2 workers, 2 farmers and ranchers, 3 others, 4 employees, and 5 self-employed. As a result, participants were classified as 3, 4, or 6 in terms of family size. Furthermore, if they were a renter or a landlord, they were assigned a score of 1 or 2. Following, each participant was assigned a score between zero and 15 for their overall SES score, and individuals were divided into three categories based on SES tertiles: low, middle, and high. Participants' physical activity levels were assessed using a shortened version of the International Physical Activity Questionnaire (IPAQ) [48]. Body composition measurements were done by the bioelectrical impedance analysis (BIA) method (Tanita, BC-418 MA, Tokyo, Japan). Height and weight were measured using a wall-mounted stadiometer and a Seca scale (Seca co., Hamburg, Germany) to the nearest 0.5 cm and 0.1 kg respectively. Waist circumference (WC) was measured at the midpoint between the lower costal margin and the iliac crest using a tape measure to the nearest 0.1 cm while hip circumference (HC) was measured over the widest part of the buttocks and was recorded to the nearest 0.1 cm. BMI and waist-to-hip ratio (WHR) were calculated. Blood pressure was measured with a standard mercury sphygmo-manometer twice in the same arm after at least 15 min of rest and then the mean of the two measurements was used for analysis. MetS was defined according to the National Cholesterol Education- Adult Treatment Panel (NCEP-ATP)- III criteria [49].

### Dietary assessments

Dietary information was collected using a validated semi-quantitative food frequency questionnaire (FFQ), adapted for the Iranian population [50]. The FFQ was a list of frequently consumed food items with specified serving sizes in Iran. The participants were asked to state whether they consumed each food item daily, weekly, monthly, or yearly, as well as how often and how much using the general standard portion sizes, cooking yields, and edible food portions provided in the Iranian household manual [51]. Each food item's reported frequency was converted to a daily intake. Using common measurements, portion amounts of consumed items were converted to grams. For instance, one slice of Taftoon bread, a typical Iranian bread, measuring 10 by 10 cm, equals 15 g.

The DRRS was calculated from nine dietary factors; diabetes- protective food items were assigned in ascending order and the diabetes-triggering factors were assigned in descending order to compose the final DRRS. Diabetes-protective food items included coffee (either caffeinated or decaffeinated), cereal fiber, nuts, whole fruits, and polyunsaturated to saturated fat ratio while the diabetes-triggering factors included GI, trans fats, SSBs/fruit juices, and meats (red and processed). As previously suggested by Kang JH et al. [40], total fruits were added as a diabetes-protective factor and combined fruit juices with SSBs as one adverse factor. For each of the nine dietary factors, we assigned for each participants a quintile value ranging from 1 (consistent with the highest type 2 diabetes risk) to 5 (consistent with the lowest type 2 diabetes risk). From the sum of these quintile values, the final DRRS is obtained. The final DRRS ranged from 9 to 45 while the higher scores denote higher adherence to the diabetes risk reduction diet.

### Biochemical assessment

10 ml venous blood samples were obtained from each individual and blood samples were centrifuged at 4500 rpm for 10 min to separate sera and plasma samples. Serum total cholesterol (TC), triglyceride (TG), high-density lipoprotein cholesterol (HDL-C), and fasting blood sugar (FBS) were evaluated using commercial kits (Pars Azmoon, Tehran, Iran). Furthermore, the low-density lipoprotein cholesterol (LDL-C) level was estimated by the Friedewald equation [26]. Enzyme-linked immunosorbent assay kits were used to measure serum insulin concentrations (Bioassay Technology Laboratory, Shanghai Korean Biotech, Shanghai City, China). Homeostatic model assessment for insulin resistance (HOMA-IR) was calculated as follows: fasting insulin (μ IU/ml) × fasting glucose (mmol/l) /22.5. Plasma agouti-related peptide (Ag-RP) and α-melanocyte-stimulating hormone (α-MSH) were assessed using enzyme-linked immunosorbent assay kits (Bioassay Technology Laboratory, China).

### Statistical analyses

Statistical analysis of the data was performed using Statistical Package for Social Sciences (version 21.0; SPSS Inc, Chicago IL) at a statistical significance level of < 0.05. Data are presented as frequency (%) for categorical variables and median ± interquartile range (IQR) for continuous variables. The differences in discrete and continuous variables across different quartiles of dietary DRRS were compared using the chi-square test and one-way ANOVA respectively. Analysis of covariance (ANCOVA) was used for the comparison of biochemical variables after adjustment for confounders (age, sex, BMI, physical activity, and energy intake). Also adjustment for mentioned potential confounders was performed for comparison of dietary intakes of study participants by dietary DRRS quartiles. Chi-square was used to test the trends of MetS’ prevalence and linear regression analysis was used for trend analysis of biochemical parameters across different DRRS quartiles. Logistic regression analysis was performed to estimate the odds ratios (ORs) and 95% confidence intervals (CIs) of the risk of MetS components across dietary DRRS quartiles after adjustment for BMI and total energy intake.

## Results

**Fig. 1:**
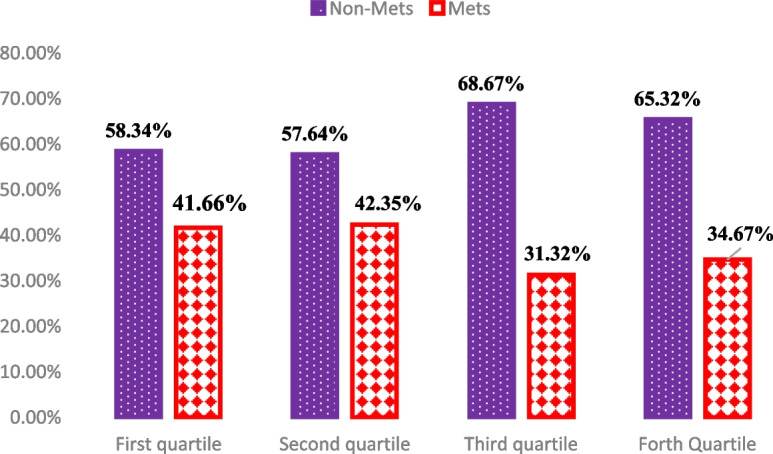
The prevalence of metabolic syndrome in different quartiles of DRRS. Mets, metabolic syndrome; DRRS, diabetes risk reduction score; Chi-square test used for test the prevalence of metabolic syndrome trend in quartiles of DRRS (*P* = 0.245). *p*-value adjusted for age, sex, BMI, physical activity, and total daily energy intake

The current cross-sectional study was conducted among 342 obese individuals (median BMI of 33.9 kg/m^2^) which includes 58% male and 42% female (median age of 38 years old). The results are presented in Tables 1, 2, 3 and 4. Table 1 shows the general characteristics of study participants according to DRRS categories. Women and those with higher BMIs were more likely to be at higher categories of DRRS. Other characteristics were not significantly different between different DRRS quartiles (*P* > 0.05). Tables 2 and 3 present the comparison of dietary energy, macronutrients and, DRRS components across different DRRS quartiles. Those with higher adherence to DRRS had a significantly higher intake of energy, and fiber compared with those at the lower quartiles. Moreover, lower intakes of trans fats, meat, SSB, and GI with higher intakes of fruits, cereal fiber, PUFA/ SFA ratio, coffee, and nuts were observed in the highest versus lowest DRRS categories (*P* < 0.001); however, after adjustment for the confounding effects of age, gender, BMI, physical activity and energy intake the significant difference of coffee, PUFA/ SFA ratio, trans fat and nuts across DRRS quartiles was lost. Higher adherence to DRRS was associated with lower systolic blood pressure (SBP) (P-adjusted = 0.01, with a remarkable reducing linear trend, 0.022), diastolic blood pressure (DBP) (P-crude = 0.012, P-trend = 0.126), TG (P-adjusted = 0.002, P-trend = 0.089) and higher HDL values (P-adjusted = 0.001, P-trend = 0.074). However, for DBP, this significant difference was disappeared after adjustment for potential confounders (P-adjusted = 0.253). No significant difference for other variables was observed. Table 5 presents odds ratios (ORs) and 95% confidence intervals (CIs) of the components of MetS risk across different quartiles of dietary DRRS after adjustment for BMI and energy intake. Subjects with higher adherence to DRRS had reduced risk of hypertension compared with those with the lower adherence (OR 0.688, 95% CI 0.26–0.84, *p* < 0.05). There was no significant association between other MetS components across DRRS quartiles. As illustrated in Fig. 1, lower prevalence of MetS was observed in the third and fourth quartiles of DRRS; although, this difference was not statistically significant (*p* = 0.245).

## Discussion

In the current study, we investigated the association between DRRS and metabolic risk factors among 342 obese individuals. According to our results, higher adherence to DRRS was associated with more favorable cardiovascular risk factors including lower blood pressure, lower TG, and higher HDL concentrations. Although, the highest DRRS quartile was associated with the lowest prevalence of Mets among obese individuals, this difference were not statistically significant after adjustment for confounders. To our knowledge, it is the first study that investigated the adherence to DRRS among obese individuals and revealed its beneficial effects toward cardio-metabolic risk factors. Although, for weight loss, different strategies have been developed and numerous interventions are available [52], but, most of obese individuals are unable to control their weight and therefore, their population is increasing worldwide [53–55]. As previously mentioned, there are a limited number of studies that evaluated adherence to DRRS and almost all of them confirmed its protective role against chronic diseases like diabetes [34], metabolic syndrome [35], and cancers [40, 41]. The ingredients of DRRS are comparable with other dietary indices; for example, higher adherence to a dietary approach to stop hypertension diet (DASH) that is full of whole grains, nuts, fruits and vegetables and, legumes was associated with lower serum lipids [56–58], lower odds of metabolic syndrome [59, 60], and more favorable cardiovascular health [61, 62] in numerous studies; a similar situation was observed for a Mediterranean dietary pattern that includes whole grains, monounsaturated fat, plant proteins, seafood, fruits, and vegetables and is associated with reduced cardiovascular risk factors [63, 64] and metabolic syndrome incidence in different populations [65, 66]. The beneficial effects of DRRS are attributed to its dietary ingredients; dietary fiber favors each MetS component separately by improving blood pressure, reducing cholesterol, improving glucose metabolism, and regulate body weight [67]. Additionally, dietary fiber reduces inflammation and oxidative stress, both of which have been associated with MetS development [68–71]. Similarly, the positive lipid-lowering effects of fruits are possibly because of their fiber, polyphenol, or phytosterol content, increased fecal bile acids and neutral steroids excretion, and increased fecal cholesterol and fatty acid excretion [72, 73]. Moreover, they exert antioxidant actions by protection against lipid peroxidation and reduced inflammatory response, and protection of vascular endothelial function [74–76]. In the study of Kempf K et al. [77], habitual coffee consumption for one month significantly reduced serum concentrations of interleukins, and increased adiponectin concentrations; also serum concentrations of total cholesterol, HDL cholesterol, and apolipoprotein A-I increased in response to regular coffee consumption among healthy subjects. Although the health effects of coffee on serum lipids and glucose tolerance depend on the type of coffee and the health status of participants; for example, in comparison to decaffeinated coffee, caffeine, ground caffeinated coffee, and instant caffeinated coffee increased lipolysis; furthermore, when compared to a placebo, acute caffeine ingestion increased glucose tolerance, whereas regular decaffeinated coffee decreased glucose tolerance [78, 79]. A meta-analysis of the intervention trials which were performed in Western countries revealed that those with hyperlipidemia were more sensitive to the cholesterol-raising effect of coffee [80]. Reduced consumption of red meat, trans fats and, SSBs are also helpful in healthy effects of DRRS. Numerous previous studies revealed the direct association between red meat consumption and blood pressure [81], twenty-year blood pressure change [82] and, incident hypertension [83]. High trans fatty acid consumption is associated with increased serum lipids and reduced consumption of trans fatty acid modifies serum lipids [84–86]. The arcuate nucleus (ARC) of the hypothalamus is one area of the brain that is a potential target for the effects of inflammatory cytokines. According to our results, the reduction in AgRP levels among DRRS quartiles was marginally significant after adjusting for confounders. Neurons in the ARC that express the neuropeptide AgRP are important areas contributing to orexigenic drive [37]. Leptin and insulin resistance develop in AgRP neurons as well as in peripheral tissues as a result of activation of the c-Jun N-terminal Kinase (JNK)-1 pathway, which also causes AgRP neurons to be activated more frequently. Therefore, JNK1 activation in AgRP neurons may cause leptin resistance, which could then lead to the development of systemic insulin resistance in obese individuals [87]. In addition, models of both acute and chronic inflammation show elevated expression of AgRP mRNA[88]. Therefore, it is expected that by increasing adherence to the DRRS and improving insulin resistance and reduced inflammation, a decrease in AgRP levels be observed; interestingly, after adjustment for the confounders, serum AgRP concentrations reduced in higher quartiles of DRRS in a marginally significant threshold (*p* = 0.065). After analysis, the most influential confounding factor in ANCOVA for the AgRP was sex, that could be explained by the regulation of AgRP expression by estrogen [89, 90]. DRRS, summarizes all of these food components altogether and identifies their synergistic or inhibitory effects same as what we encounter in our daily usual diet. So, it is a unique indicator of healthy dietary intake and the its effects are more realistic compared with other food scores with a very limited number of food ingredients. The current study has also some limitations; first of all, the cross-sectional design of the study limits the casual inference. Second, there was no long-term follow-up of study participants and due to dynamic change of both diet (e.g. DRRS) and cardio-metabolic risk factors over the years, longitudinal assessment of these factors will help to explain the causality. Third, the FFQ that was used in the current study was not originally developed for DRRS evaluation and the collected data may stem for recall bias, however, we used a valid and reliable FFQ that is adapted for the target population and we performed this study in a relatively large number of participants.

In conclusion, in the current cross-sectional study, DRRS was associated with favorable blood pressure and serum lipids in obese individuals. Moreover, the lowest prevalence of MetS was observed in the highest DRRS categories. Due to the limited number of studies investigating the health benefits of DRRS, further studies are warranted to identify its health effects in different populations and different disease statuses.

**Table 1:** General characteristics of study population by dietary DRRS quartiles

Variable	**All participants** (***N*** = 342)	**Quartiles of DRRS**				
		**1**^**st**^ ^(***N***=86)^	**2**^**nd**^ ^(***N***=85)^	**3**^**rd**^ ^(***N***=85)^	4^th^ ^(***N***=86)^	***P*** * **value**
	**Median** **(IQR)**	**Median (IQR)**	**Median (IQR)**	**Median (IQR)**	**Median (IQR)**	
Age (y)	38 (32.00–44.00)	34.50 (32.50–47.50)	39.00 (34.00–48.00))	41.00 (36.00–47.50)	36.00 (33.00–48.00)	0.497
Sex (% Male)	58	74	51	36	36	**0.001**
WC (cm)	110 (103.00–114.00)	111.50 (107.00–116.75)	107.00 (102.00–112.50)	110.00 (103.00–114.00)	108.00 (101.00–116.00)	0.760
BMI (kg/m^2^)	33.99 (31.87–37.05)	33.21 (32.14–37.41)	33.60 (31.18–34.71)	35.01 (32.25–36.87)	33.99 (31.65–37.52)	**0.002**
SES score	10 (8.00–12.00)	11.00 (9.00–13.00)	10.00 (8.00–12.00)	10.00 (8.00–11.00)	9.00 (7.00–11.00)	0.074
FM (%)	32.80 (26.80–39.70)	29.70 (27.10–40.40)	31.40 (25.30–38.40)	35.60 (28.35–38.80)	34.40 (26.6–40.80)	0.543
FFM (%)	62.70 (50.60–73.70)	69.85 (53.80–77.17)	63.50 (50.55–72.80)	59.10 (50.80–73.40)	53.70 (49.55–72.85)	0.154
BMR (Kcal)	1920 (1519.00–2184.00)	2080.00 (1685.50–2376.25)	1878.10 (1577.50–2127.00)	1826.95 (1475.00–2123.50)	1702.00 (1466.00–2137.50)	0.210
PA (MET-min/week)	830 (231.00–2658.00)	721.00 (214.50–2027.00)	1050.00 (322.00–4029.00)	918.00 (318.00–3354.00)	735.00 (82.50–2196.00)	0.390

Data represented as median (interquartile 25–75) except gender, that is presented as the percentage of males in each quartile, *DRRS* diabetes risk reduction score, *IQR* interquartile range, *BMI* body mass index, *WC* waist circumference, *BMI* body mass index, *FM* fat mass, *FFM* fat free mass, *BMR* basal metabolic rate, *PA* physical activity; all data are mean (± SD) except gender, that is presented as the percent of males in each quartile. P* values derived from Kruskal–Wallis analysis

**Table 3:** The comparison of energy and macronutrient intakes of study population by dietary DRRS quartiles

Variable	Quartiles of DRRS					
	**1**^**st**^ ^(***N***=86)^	**2**^**nd**^ ^(***N***=85)^	**3**^**rd**^ ^(***N***=85)^	4^th^ ^(***N***=86)^	P* **value**	P** **value**
	**Median (IQR)**	**Median (IQR)**	**Median (IQR)**	**Median (IQR)**		
**Energy (kcal/d)**	2603.65 (1995.59–3191.69)	2451.38 (2075.59–3255.68)	2979.82 (2183.79–3516.77)	3426.47 (2777.74–4370.09)	***P*** ** < 0.001**	***P*** ** < 0.001**
**CHO (%)**	58.71 (50.87–62.30)	57.26 (51.18–61.91)	60.63 (54.90–64.36)	57.84 (53.02–63.53)	0.181	0.529
**Protein (%)**	13.62 (12.37–14.64)	13.34 (11.85–14.42)	13.28 (11.57–14.58)	12.30 (11.27–13.62)	0.073	0.438
**Fat (%)**	30.05 (25.56–37.96)	31.46 (27.32–37.95)	28.30 (25.54–35.16)	31.75 (26.87–38.12)	0.258	0.472

Data represented as median (interquartile 25–75), *DRRS* diabetes risk reduction score, *IQR* interquartile range, *CHO* carbohydrate; *P*- values derived from One-Way ANOVA with Tukey’s post-hoc comparisons.*crude *P* values, **P values after adjustment for confounders (age, gender, BMI, physical activity and energy intake)

**Table 4:** The comparison of DRRS and its components by dietary DRRS quartiles

Variable	Quartiles of DRRS				P* value	P** value
	**1**^**st**^ ^**(*N*=86)**^	**2**^**nd**^ ^**(*N*=85)**^	**3**^**rd**^ ^**(*N*=85)**^	4^th^ ^**(*N*=86)**^		
	**Median (IQR)**	**Median (IQR)**	**Median (IQR)**	**Median (IQR)**		
**DRRS**	21.00 (20.00–22.75)	25.00 (24.00–26.00)	28.00 (27.00–29.00)	32.50 (30.75–34.00)	***P*** ** < 0.001**	0.027
**GI**	48.56 (45.67–52.16)	47.39 (43.33–50.84)	45.42 (42.35–47.81)	44.25 (41.83–46.23)	***P*** ** < 0.001**	0.009
**Coffee (g/d)**	0.95 (0.00–9.33)	1.53 (0.00–11.66)	4.66 (0.00–38.05)	4.66 (0.00–40.00)	**0.002**	0.178
**Cereal fiber (g/d)**	45.06 (32.27–57.13)	50.37 (34.26–69.72)	65.43 (43.66–95.22)	87.49 (59.98–120.64)	***P*** ** < 0.001**	***P*** ** < 0.001**
**PUFA/ SFA ratio**	0.63 (0.50–0.84)	0.77 (0.65–0.92)	0.80 (0.69–1.01)	0.98 (0.80–1.29)	***P*** ** < 0.001**	0.080
**SSBs (g/d)**	7.30 (2.42–17.55)	2.19 (0.13–7.87)	2.79 (0.00–19.03)	0.00 (0.00–2.46)	***P*** ** < 0.001**	***P*** ** < 0.001**
**Trans (g/d)**	0.16 (0.05–0.87)	0.06 (0.00–0.32)	0.05 (0.00–0.66)	0.00 (0.00–0.05)	***P*** ** < 0.001**	0.708
**Fruits (g/d)**	299.54 (207.37–470.43)	369.04 (222.58–478.04)	559.85 (362.87–823.52)	497.64 (339.37–854.06)	***P*** ** < 0.001**	***P*** ** < 0.001**
**Nuts (g/d)**	8.20 (0.88–6.56)	7.71 (1.69–7.10)	14.47 (2.46–11.90)	18.44 (1.67–15.53)	***P*** ** < 0.001**	0.826
**Meat (g/d)**	15.90 (14.64–23.19)	16.61 (13.18–27.05)	13.71 (7.72–15.25)	14.33 (13.38–17.03)	0.014	***P*** ** < 0.001**

Data represented as median (interquartile 25–75), *DRRS* diabetes risk reduction score, *IQR* interquartile range, *GI* glycemic index, *PUFA* polyunsaturated fatty acids, *SFA* saturated fatty acids, *SSBs* sugar-sweetened beverages; *P*- values derived from One-Way ANOVA with Tukey’s post-hoc comparisons. *crude *P* values, **P values after adjustment for confounders (age, gender, BMI, physical activity and energy intake)

**Table 2:** Biochemical parameters of study population by dietary DRRS quartiles

**Variable**	**Quartiles of DRRS**						
	**1**^**st**^ ^(***N***=86)^	**2**^**nd**^ ^(***N***=85)^	**3**^**rd**^ ^(***N***=85)^	4^th^ ^(***N***=86)^	P* **value**	P** **value**	***P***- **Trend** ***
	**Mean (SD)**	**Mean (SD)**	**Mean (SD)**	**Mean (SD)**			
**SBP (mmHg)**	126.79 (15.59)	122.20 (14.84)	122.12 (13.81)	118.87 (20.22)	**0.013**	**0.010**	**0.022**
**DBP (mmHg)**	84.59 (10.87)	80.69 (10.59)	81.90 (10.36)	79.08 (14.37)	**0.012**	0.253	0.126
**FBS (mg/dl)**	92.69 (25.42)	92.02 (14.76)	94.53 (16.12)	91.88 (17.40)	0.822	0.125	0.241
**TC (mg/dl)**	194.27 (34.43)	191.40 (42.22)	192.36 (36.90)	187.56 (32.97)	0.677	0.432	0.115
**TG (mg/dl)**	174.38 (92.21)	151.52 (119.09)	152.69 (95.91)	122.87 (50.11)	**0.004**	**0.002**	0.089
**HDL (mg/dl)**	41.01 (8.64)	45.32 (10.00)	42.48 (9.31)	45.44 (9.59)	**0.002**	**0.001**	0.074
**LDL (mg/dl)**	124.02 (31.16)	123.78 (33.46)	125.72 (31.98)	120.00 (31.47)	0.720	0.581	0.345
**Insulin (Miu/L)**	17.19 (18.88)	14.67 (10.47)	17.46 (10.48)	15.56 (11.96)	0.647	0.104	0.146
**HOMA-IR**	4.01 (4.43)	3.47 (2.83)	4.15 (2.71)	3.50 (2.55)	0.574	0.129	0.198
**α- MSH (ng/L)**	231.88 (177.85)	173.21 (130.88)	242.15 (175.18)	224.46 (175.39)	0.211	0.226	0.423
**AgRP (Pg/ml)**	33.14 (21.28)	27.74 (15.18)	34.17 (20.03)	29.50 (18.31)	0.324	0.065	0.669

*SBP* systolic blood pressure, *DBP* diastolic blood pressure, *TC* total cholesterol, *TG* triglyceride; HDL-C, high density lipoprotein cholesterol; LDL-C, low density lipoprotein cholesterol; HOMA-IR, homeostatic model of insulin resistance; α-MSH, melanocyte stimulating hormone; AgRP, agouti-related peptide; P* values for crude and P** values derived from ANCOVA after adjustment for confounders (age, gender, BMI, physical activity and energy intake). *** *P* trends derived from linear regression test after adjustment for potential confounders

**Table 5:** Odd’s ratio (OR) and confidence interval (CI) for metabolic syndrome components risk by dietary DRRS quartiles

**MetS components**	**Quartiles of DRRS**				P for trend
	**1**^**st**^ ^(***N***=86)^	**2**^**nd**^ ^(***N***=85)^	**3**^**rd**^ ^(***N***=85)^	4^th^ ^(***N***=86)^	
High WC	Ref	0.950 (0.13–3.80)	0.683 (0.08–2.84)	1.32 (0.20–5.70)	0.162
Hyperglycemia	Ref	0.528 (0.18–1.53)	0.441 (0.15–1.30)	0.398 (0.13–1.18)	0.734
Hypertension	Ref	**0.231** **(0.06–0.87)**	**0.789** **(0.26–0.92)**	**0.688** **(0.26–0.84)**	0.092
High TG	Ref	0.517 (0.17–1.56)	0.629 (0.21–1.86)	0.486 (0.16–1.46)	0.143
Low HDL-C	Ref	0.560 (0.22–1.40)	1.152 (0.45–2.90)	1.138 (0.45–2.84)	0.136

The multivariate multinomial logistic regression was used for the estimation of ORs and confidence interval (CI) after adjustment for confounders (BMI and energy intake). *MetS* metabolic syndrome, *WC* waist circumference, *HDL-C* high-density lipoprotein cholesterol, *T*G triglyceride. High WC, WC ≥ 88 cm in females and ≥ 102 cm in males; Hyperglycemia, fasting glucose ≥ 100 mg/dl; Hypertension, DBP ≥ 85 mmHg or SBP ≥ 130 mmHg; High TG, TG ≥ 150 mg/dl; Low HDL-C, HDL-C < 50 mg/dl in females and < 40 mg/dl in males. Bold values show statistically significant threshold (*P* < 0.05)

## Data Availability

The datasets generated and/or analyzed during the current study are not publicly available due to some restrictions that applied by the ethical committee but are available from the corresponding author on reasonable request.
